# Adapted Basketball Training Improves Fitness and Motivation in Adolescents with Moderate Obesity: A Randomized Controlled Trial

**DOI:** 10.3390/children12091262

**Published:** 2025-09-19

**Authors:** Oumayma Slimi, Mohamed Abdelkader Souissi, Santo Marsigliante, Georgian Badicu, Jolita Vveinhardt, Antonella Muscella

**Affiliations:** 1 High Institute of Sport and Physical Education, University of Sfax, Sfax 3029, Tunisia; oumayma.slimii@gmail.com; 2 Physical Activity, Sport and Health Research Unit, UR18JS01, National Observatory of Sport, Tunis 1003, Tunisia; gaddoursouissi@yahoo.com; 3 Department of Biological and Environmental Sciences and Technologies (Di.S.Te.B.A.), University of Salento, 73100 Lecce, Italy; santo.marsigliante@unisalento.it; 4 Department of Physical Education and Special Motricity, Faculty of Physical Education and Mountain Sports, Transilvania University of Braşov, 500036 Braşov, Romania; georgian.badicu@unitbv.ro; 5 Institute of Sport Science and Innovations, Lithuanian Sport University, 44221 Kaunas, Lithuania

**Keywords:** obesity, physical fitness, motivation, basketball training, video-based instruction

## Abstract

**Background:** Adolescent obesity represents a global public health issue, with an urgent need for tailored intervention strategies, particularly in school settings. **Objectives:** This study investigated the impact of adapted basketball training—with and without video summaries—on BMI, fitness, motivation, and technical skills in adolescents with moderate obesity. **Methods:** Fifty-five participants were randomly assigned to one of three instructional modalities—a modified basketball program enriched with video summaries (ADAPT + VID), a modified program without videos (ADAPT), and a traditional program (CONT)—and completed an eight-week intervention. Before (T0) and after (T1) the intervention, participants in all three groups underwent testing to assess: (i) anthropometric measurements (BMI), (ii) technical accuracy (passing, dribbling, and shooting), (iii) physical fitness using the Intermittent Fitness Test 15-15 (Spartacus Test), and (iv) motivation using the Situational Motivation Scale. **Results:** Assessments revealed significant improvements in the ADAPT + VID group, with notable reductions in BMI (d = 0.25, *p* < 0.05), enhanced physical fitness (d = 0.19, *p* = 0.002), and improved performance in passing (d = 1.42, *p* < 0.001), dribbling (d = 1.30, *p* < 0.001), and free-throw shooting (d = 0.80, *p* < 0.001). Moreover, a significant increase in intrinsic motivation (d = 1.18, *p* < 0.001) and a reduction in amotivation (d = 1.12, *p* < 0.001) were observed. **Conclusions:** These findings suggest that incorporating pedagogical video summaries into an adapted basketball program may effectively improve physical health, motor skills, and motivation among adolescents with moderate obesity.

## 1. Introduction

Adolescent obesity has reached alarming proportions, evolving from a localized health concern into a global epidemic [1,2]. According to the latest estimates from the World Health Organization, the global prevalence of obesity among adolescents has tripled over the past three decades, with nearly 18% of adolescents now affected by overweight or obesity. Previous research has shown that approximately 80% of adolescents with obesity maintain their excess weight into adulthood, particularly if lifestyle habits remain unchanged [3,4]. This escalation exposes an entire generation to heightened risks of chronic diseases [2] and psychosocial disorders [5]. Even more concerning is that adolescents with obesity are affected not only by poor dietary habits or physical inactivity but also by the lack of evidence-based and developmentally appropriate physical activity interventions [6]. Physical inactivity and sedentary lifestyle are among the major factors contributing to the etiology of obesity [7]. However, the study of physical activity promotion in the clinical management of obesity is yet to be investigated to guide optimized interventions that can be fully embraced by physically reluctant youth [6,8].

Recently, different physical activity modalities have been suggested to reduce the prevalence of obesity [6,9,10] while improving psychological indicators. The inclusion of obese adolescents in physical education (PE) classes represents a viable intervention setting, since young people spend a large part of their time at school. However, PE classes represent a major challenge due to multiple physiological, psychological, and social barriers that hinder their active participation [11].

Addressing these issues requires pedagogical approaches specifically designed to promote engagement [11,12] and improve motor skills [10]. Among the most effective strategies to enhance participation and well-being among students with obesity are three approaches with proven effectiveness. These include the Teaching Games for Understanding (TGFU) model, the use of structured verbal encouragement, and the implementation of adapted physical education programs [11,13,14].

The TGFU model represents a pedagogical approach that focuses on understanding tactical and decision-making skills in games, rather than practicing individual technical skills [15].

By offering contextualized, game-like situations tailored to participants’ abilities, this model promotes an optimal motivational climate, reduces performance-related anxiety, and stimulates both motor and social engagement [16,17]. Its relevance for students with obesity lies in its ability to reframe expectations in PE around enjoyment and inclusion, thus mitigating the stigma often associated with presumed physical incompetence.

Another key lever for optimizing the PE experience of obese learners involves the strategic use of structured verbal encouragement by physical education teachers (PETs). When delivered intentionally, these forms of positive reinforcement have a direct impact on students’ psychophysiological and motivational responses. By focusing on effort rather than outcome and emphasizing perceived competence, verbal encouragement significantly enhances self-efficacy—a core determinant of sustained engagement in physical tasks [18].

Empirical evidence shows that students exposed to supportive verbal feedback demonstrate greater persistence in the face of motor challenges, better stress regulation, and a reduction in negative affect related to body image [19,20]. Furthermore, regular encouragement from PETs contributes to the creation of a caring and inclusive learning environment—an essential condition for maximizing the engagement of adolescents with obesity and fostering long-term adherence to physical activity [21,22].

Recently, Slimi et al. [6] showed that an adapted basketball cycle reduced perceived difficulty by 27% in boys and 36% in girls (*p* < 0.001), while girls also reported a 27% increase in enjoyment (*p* < 0.001). These findings emphasize the value of tailored adaptations in promoting adherence, particularly among female adolescents with overweight.

Taken together, these findings emphasize the imperative for PE teachers to adopt differentiated instructional strategies that address the specific needs of adolescents with obesity, in order to optimize their engagement and well-being through appropriately tailored physical activity.

Nevertheless, Crane and Temple [23] reported that conventional interventions often suffer from high dropout rates, primarily due to lack of enjoyment, low perceived competence, social pressures, competing priorities, and physical limitations. In this context, the integration of video technology into PE programs has emerged as an innovative pedagogical strategy to overcome such barriers [24,25]. Indeed, video use enriches the learning experience by making activities more interactive and immersive, thereby promoting student engagement and motivation [26,27]. Moreover, it enhances the structuring of instructional content through accurate demonstrations [28], in-depth movement analyses, and visual materials tailored to learners’ specific needs [29]. Easy access to dynamic digital resources also fosters autonomy and self-assessment skills [28,29], which, in turn, strengthen students’ perceived competence and active involvement in the learning process [30]. Therefore, the systematic integration of video technology in physical and sports education may serve as a promising strategy to enhance learning and motivation among adolescents, particularly those facing challenges such as obesity, by making sessions more accessible, engaging, and personalized.

The objective of this study is to examine the effects of three intervention modalities on changes in body mass index (BMI), physical fitness, motivation, and technical performance in basketball among adolescents with obesity. The three modalities include: (i) an adapted intervention program supplemented with pre-session video summaries of instructional content (ADAPT + VID), (ii) an adapted intervention program without video supplementation (ADAPT), and (iii) a traditional basketball program without specific pedagogical modifications (CONT). We hypothesize that integrating video summaries into the adapted intervention program (ADAPT + VID) will lead to significantly greater improvements compared to both the adapted-only (ADAPT) and traditional (CONT) programs:

• H1: Greater reduction in BMI
• H2: Greater improvements in physical fitness
• H3: Higher levels of motivation
• H4: Enhanced technical execution of basketball skills

## 2. Materials and Methods

### 2.1. Participants

Based on an a priori power analysis conducted using G*Power 3.1.9.4 (University of Düsseldorf, Germany), a total sample size of 54 participants (18 per group) was required to detect a medium effect size (Cohen’s f = 0.25) for a mixed-model ANOVA with three groups and two time points (Group × Time interaction), assuming α = 0.05 and statistical power (1 − β) = 0.80. This anticipated effect size aligns with prior school-based intervention studies involving adolescents with obesity aged 14–17 years that yielded moderate-to-large improvements in BMI, motor skills, and fitness [31,32].

Prior to the start of the study, participants and their legal guardians received a detailed explanation of the study’s objectives and experimental procedures. Written informed consent was obtained from all participants and their parents or guardians. The study protocol adhered to the ethical principles outlined in the Declaration of Helsinki (2013) for research involving human participants and was approved by the Ethics Committee of the Faculty of Medicine of Sfax (Approval No. 048/2022).

A total of 66 adolescents were initially recruited from a school in the Sidi Bouzid region of Tunisia. Of these, 59 met the eligibility criteria and were randomly assigned to one of three groups (ADAPT, ADAPT + VID, Control). During the intervention, four participants withdrew (three due to illness and one for personal reasons). Thus, 55 participants (33 females and 22 males), classified as having moderate obesity, completed the intervention and were included in the final analyses.

**Figure 1:**
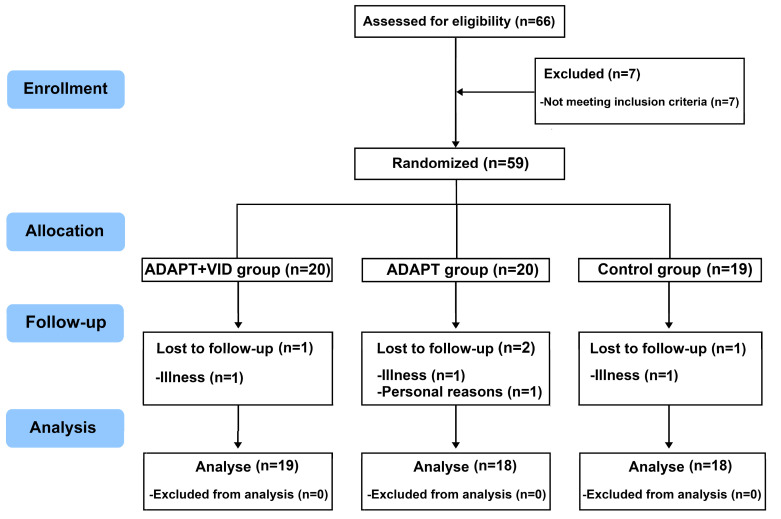
Flow-chart of study.

After baseline assessments of technical basketball skills, physical fitness, and BMI, 66 adolescents were initially recruited from a school in the Sidi Bouzid region of Tunisia. Of these, 59 met the eligibility criteria (age 15–17 years; BMI 30–34.9 kg/m^2^; no musculoskeletal, neurological, or orthopedic disorders in the previous six months; normal or corrected-to-normal vision; regular attendance in PE classes) and were randomly assigned using a computer-generated sequence to one of three groups: (i) an adapted intervention program enriched with pre-session video summaries (ADAPT + VID; n = 19 [11 females, 8 males]); (ii) an adapted program without video supplementation (ADAPT; n = 18 [11 females, 7 males]); and (iii) a traditional basketball program (CONT; n = 18 [11 females, 7 males]). Four participants withdrew during the intervention (three due to illness, one for personal reasons), leaving 55 adolescents who completed the study. Independent *t*-tests and Mann–Whitney U tests confirmed no significant differences between groups at baseline. Figure 1 presents the enrollment, allocation, intervention, and analysis phases according to the CONSORT flow diagram.

### 2.2. Procedure

This study is a three-arm, parallel-group randomized controlled trial conducted in accordance with the CONSORT guidelines.

One week before the experimental phase, participants were familiarized with the experimental procedures, testing protocols, and equipment to minimize learning effects during the study.

All three participant groups completed testing sessions designed to assess (i) technical performance of basketball skills, including passing, dribbling, and free-throw shooting; (ii) physical fitness; and (iii) body mass index (BMI), at two time points: before the intervention (T0) and after (T1) a basketball teaching unit consisting of seven sessions. Additionally, motivation levels were evaluated after the first and last basketball sessions.

Testing sessions were scheduled during regular physical education classes, between 10:00 a.m. and 12:00 p.m.

Each group followed a specific instructional program according to the assigned intervention modality. The program consisted of one weekly session over eight consecutive weeks. Each session lasted one hour and was structured into three distinct phases: (i) a warm-up phase, (ii) a main learning and practice phase, and (iii) a cool-down phase.

The warm-up phase, common to all groups, included upper and lower limb exercises and preparatory games, lasting 15 min.

During the 40 min main phase, participants engaged in basketball exercises adapted to their respective intervention conditions:

Adapted program enriched with pre-session video summaries (ADAPT + VID): Participants in this group were given access to a private Facebook platform, where video summaries of instructional content—focusing on basketball techniques such as passing, dribbling, and free-throw shooting—were posted 48 h before each physical education session. The Facebook “Seen by” feature allowed researchers to monitor participants’ engagement with the videos and identify those who had not viewed the materials. Personalized reminders were sent to ensure equitable access to instructional resources and enhance learner engagement. All participants and their legal guardians provided written informed consent for the use of digital platforms, and access to the content was restricted to study participants and research staff to protect privacy and confidentiality.

Adapted program only (ADAPT): Participants in this group completed only the pedagogical activities specified in the adapted intervention program without supplementary video content.

Control group (CONT): Participants followed a traditional basketball program consisting of the same number of sessions as the experimental groups but without any pedagogical modifications.

The cool-down phase lasted 5 min and consisted of relaxation exercises aimed at promoting physiological and psychological recovery.

### 2.3. Adapted Basketball Program

The adapted basketball program was based on the protocol proposed by Slimi et al. [6], designed to provide inclusive, enjoyable, and adapted activities to adolescents with obesity, minimizing competitive elements that could potentially induce anxiety or disengagement.

The intervention incorporated several adaptations into the game of basketball to provide adolescents with obesity more time and space to gain confidence and improve decision-making. For example, defenders were limited to using only one hand and were asked to delay their interventions until the attacker completed the pass [33].

Similarly, traditional 3-on-3 drills were restructured into asymmetric 3-on-2 formats to increase attacking opportunities while facilitating the learning of tactical principles in a less demanding environment [33,34]. Additionally, regular offensive rotations were scheduled throughout the sessions, allowing participants to benefit from frequent recovery periods, which are necessary for the physiological demands of adolescents with obesity [33].

The detailed program structure is presented in Table 1.

### 2.4. Anthropometric Measurement

Accurate anthropometric assessments were conducted for all participants. Body weight was measured to the nearest 0.1 kg using a portable digital scale (Tanita BC-533, Tokyo, Japan). and height was recorded to the nearest millimeter using a portable Holtain stadiometer (Holtain Ltd., Crymych, UK), with participants standing barefoot or in socks. BMI was calculated using the standard formula: body weight (kg) divided by height squared (m^2^).

### 2.5. Intermittent Fitness Test 15-15 (Spartacus Test)

The Spartacus test is an aerobic intermittent shuttle run designed to assess cardiorespiratory fitness in children and adolescents with obesity or overweight [35]. Participants perform repeated 15 s runs over 40 m, with 15 s passive recovery intervals. The running speed starts at 5 km/h and increases by 0.5 km/h after each interval, guided by pre-recorded audio signals. The test ends when participants cannot maintain their pace or stop voluntarily. Total distance covered provides an indirect estimate of maximum aerobic capacity (VO_2_peak). The test demonstrated high reliability (ICC > 0.90) and strong construct validity in this population. .

### 2.6. Technical Accuracy Assessment

The technical accuracy of participants in basketball-specific motor performances was assessed using standardized tests recognized for their validity and reliability in evaluating sports skills. Three fundamental abilities were examined: passing accuracy, shooting accuracy, and dribbling proficiency. Tests were conducted on a basketball court in a gym. All tests were supervised by a trained professional to ensure inter-rater reliability. Instructions given to participants were identical in all groups, and scores were recorded anonymously for subsequent statistical analysis.

#### 2.6.1. Passing Accuracy

**Figure 2:**
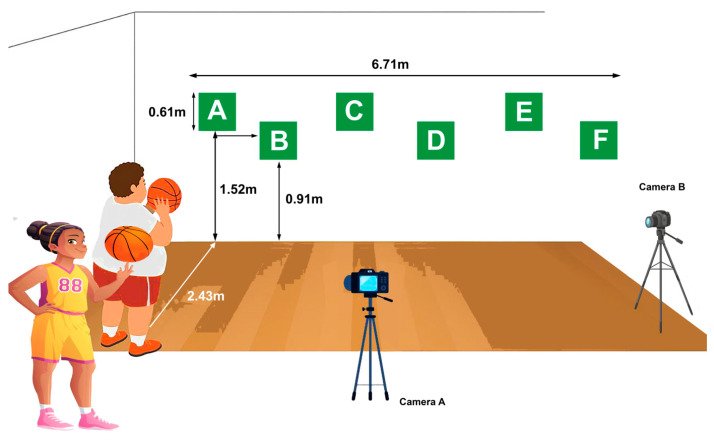
Diagram of the passing test setup, illustrating the key components: targets labeled A through F; Camera A aimed at the targets to capture pass accuracy, and Camera B positioned to record movement duration. Modified by [37].

Passing accuracy and speed were evaluated using a polygonal passing test (Figure 2), with a 30 s duration limit. Scoring awarded 2 points for direct hits, 1 point for near misses, and 0 points for violations. The test included one training trial and two scored trials, with the sum constituting the final score. This protocol has been validated previously, showing high test–retest reliability (r = 0.84–0.97) [36,37].

#### 2.6.2. Shooting Accuracy

Shooting performance was assessed using a standardized free throw test consisting of 10 consecutive shots from the free throw line (4.57 m), using a size-6 ball in accordance with FIBA recommendations. Performance was scored with a validated six-level scale [38], and the total score was obtained by summing the points across all attempts.

#### 2.6.3. Dribbling Mastery

Dribbling mastery, agility, and speed were assessed using a standardized zigzag dribbling course with five cones spaced 2 m apart. Participants completed two trials, and the fastest time was retained for analysis [39,40]. Performance time was measured with a digital stopwatch (0.01 s accuracy).

### 2.7. Motivation Assessment

During sessions 1 and 7, participants’ motivation was measured using the Situational Motivation Scale (SIMS), validated to assess different forms of motivation in specific situations [41]. SIMS distinguishes four subdimensions, intrinsic motivation, identified regulation, external regulation and amotivation, measured through four items: intrinsic motivation (items 1, 5, 9, 13), identified regulation (items 2, 6, 10, 14), external regulation (items 3, 7, 11, 15) and amotivation (items 4, 8, 12, 16). Responses were collected using a 7-point Likert scale, from 1 (“does not correspond at all”) to 7 (“corresponds exactly”), allowing for a precise detection of the intensity of motivation perceived by participants. Internal reliability was confirmed with Cronbach’s alpha values of α = 0.82–0.91 across subscales, indicating good to excellent consistency.

### 2.8. Statistical Analyses

Statistical analyses were performed using Statistica 10 software (StatSoft, Krakow, Poland). The Shapiro–Wilk test confirmed that all variables satisfied the normality assumption and the Levene test confirmed the homogeneity of the data, supporting the suitability of the ANOVA model for intergroup comparisons. To analyze the changes in the parameters under examination, a repeated measures ANOVA was conducted involving three groups (ADAPT + VID, ADAPT, and CONT) and two measurement periods (before [T0] and after intervention [T1]). Effect sizes were calculated using partial eta squared (η^2^p) for all ANOVA results and interpreted as small (0.01), medium (0.06), or large (0.14). Post hoc comparisons were performed applying the Bonferroni correction to identify significant mean differences between groups. Additionally, Cohen’s d was calculated for all pairwise comparisons, with interpretation according to Cohen’s classification: small (d ≥ 0.2), medium (d ≥ 0.5), and large (d ≥ 0.8). Statistical significance was set at *p* < 0.05.

## 3. Results

### 3.1. Body Mass Index

**Figure 3:**
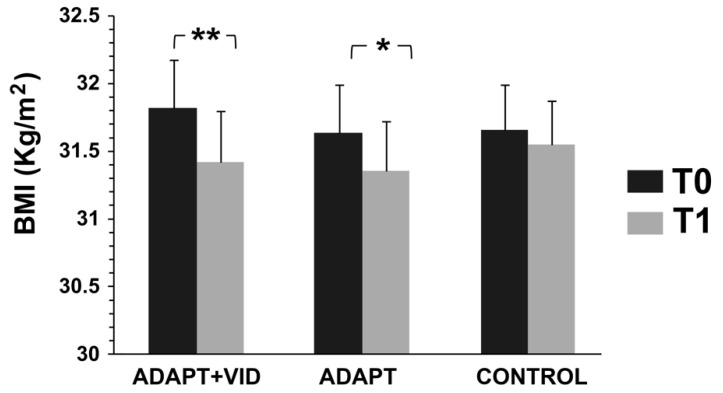
Body Mass Index (BMI) measured before (T0) and after intervention (T1) for the three groups: Control, ADAPT, and ADAPT + VID. ** *p* < 0.001; * *p* < 0.05 by Bonferroni post hoc tests.

BMI decreased significantly at T1 in both intervention groups, but participants in the ADAPT + VID group achieved the greatest benefits. In detail, a mixed-model analysis of variance (ANOVA) [(3 Groups × 2 Time Points)] with repeated measures on the second factor (see Figure 3) yielded the following results: (i) a significant main effect of time on BMI [F(1, 52) = 34.65, *p* < 0.001, η^2^p = 0.40] and (ii) a significant group × time interaction effect [F(2, 52) = 3.55, *p* < 0.05, η^2^p = 0.12], suggesting that the extent of BMI changes varied across groups.

Post hoc comparisons revealed a significant reduction in BMI from T0 to T1 in the ADAPT + VID group (Δ = 1.27 ± 0.75%, d = 0.25), demonstrating a small effect size. Similarly, in the ADAPT group, post hoc analyses confirmed a significant decrease in BMI from pretest to posttest (Δ = 0.88 ± 1.22%, d = 0.18), though with a smaller effect size.

### 3.2. Physical Fitness

**Figure 4:**
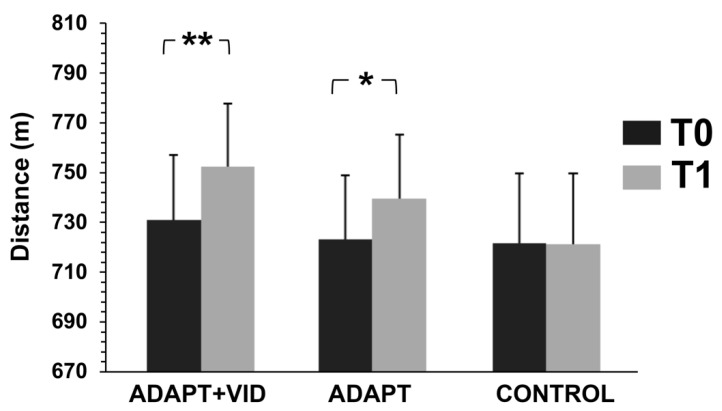
Physical Fitness measured before (T0) and after intervention (T1) for the three groups: Control, ADAPT, and ADAPT + VID. ** *p* < 0.001; * *p* < 0.05 by Bonferroni post hoc tests.

Physical fitness significantly improved over time [F(1, 52) = 17.78, *p* < 0.001, η^2^p = 0.25], with a significant group × time interaction indicating that the magnitude of improvement varied between groups [F(2, 52) = 4.90, *p* < 0.05, η^2^p = 0.15. Post hoc comparisons revealed that physical fitness significantly increased from pre- to post-test in the ADAPT + VID group (*p* = 0.002, d = 0.19) and in the ADAPT group (*p* < 0.05, d = 0.14) (Figure 4).

### 3.3. Technical Accuracy

#### 3.3.1. Passing Performance

All participants showed improvements over time [F(1, 52) = 18.94, *p* < 0.001, η^2^p = 0.27]. A mixed-model ANOVA revealed a non-significant main effect of group [F(2, 52) = 3.07, *p* = 0.05, η^2^p = 0.11], likely because the overall group comparison includes all groups and does not account for specific pairwise differences. In contrast, a significant group × time interaction effect was observed [F(2, 52) = 3.97, *p* < 0.05, η^2^p = 0.18], indicating that changes over time differed among groups.

**Figure 5:**
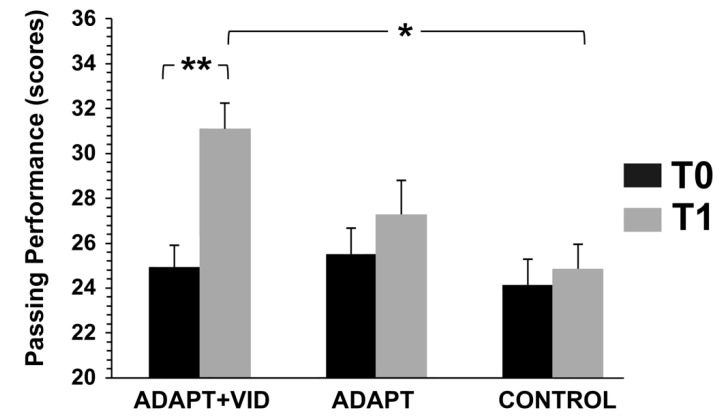
Changes in passing performance measured before (T0) and after intervention (T1) for the three groups: Control, ADAPT, and ADAPT + VID. A significant main effect of time and a significant group × time interaction was observed. ** *p* < 0.001; * *p* < 0.05 by Bonferroni post hoc tests.

Post hoc comparisons clarified these differences: the ADAPT + VID group showed the greatest improvement (Δ = 26.33 ± 16.65%, d = 1.42) and performed significantly better than the control group at T1 (*p* = 0.003, d = 1.32) (Figure 5. These findings suggest that the combined intervention, integrating the ADAPT approach with video support, was particularly effective.

#### 3.3.2. Free Throw Performance

**Figure 6:**
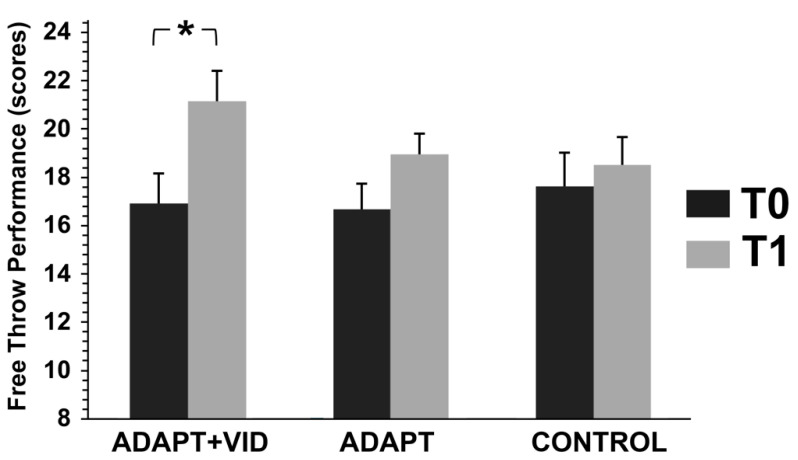
Changes in Free Throw Performance before (T0) and after intervention (T1) for the three groups: Control, ADAPT, and ADAPT + VID. A significant main effect of time and a significant group × time interaction was observed. * *p* < 0.05 by Bonferroni post hoc tests.

A mixed-design ANOVA [(3 groups × 2 time points)] with repeated measures on the second factor (see Figure 6) revealed: (i) a highly significant main effect of time on shooting performance [F(1, 52) = 27.34, *p* < 0.001, η^2^p = 0.34], (ii) a non-significant main effect of group [F(2, 52) = 0.35, *p* = 0.7, η^2^p = 0.02], and (iii) a significant interaction effect between group and time [F(2, 52) = 4.32, *p* < 0.05, η^2^p = 0.14].

Post hoc analyses indicated that, in the ADAPT + VID group, shooting performance significantly improved in T1 (Δ = +29.27 ± 22.16%, d = 0.8).

#### 3.3.3. Dribbling Performance

**Figure 7:**
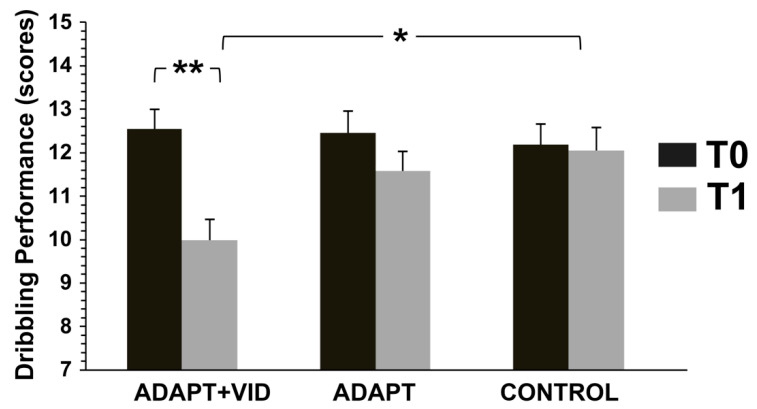
Changes in Dribbling Performance before (T0) and after intervention (T1) for the three groups: Control, ADAPT, and ADAPT + VID. A significant main effect of time and a significant group × time interaction was observed. ** *p* < 0.001; * *p* < 0.05 by Bonferroni post hoc tests.

There was an overall improvement in dribbling performance over time (main effect of time) [F(1, 52) = 22.44, *p* < 0.001, η^2^p = 0.30]; therefore, considering all groups together, performance improved significantly from T0 to T1. The improvement of ADAPT + VID was greater than that of the ADAPT group [F(2, 52) = 8.38, *p* < 0.001, η^2^p = 0.24]. No improvement was observed in the control group. Non-significant main effects of group [F(2, 52) = 1.25, *p* = 0.29, η^2^p = 0.06] were revealed (Figure 7).

Post hoc analyses, corrected for multiple comparisons using Bonferroni, revealed that dribbling performance in the ADAPT + VID group significantly improved at T1 (Δ = 19.30 ± 16.25%, d = 1.30).

### 3.4. Motivation

Intrinsic motivation showed a significant main effect of time [F(1, 52) = 26.49, *p* < 0.001, η^2^p = 0.34], a significant main effect of group [F(2, 52) = 3.72, *p* = 0.03, η^2^p = 0.13], and a significant group × time interaction [F(2, 52) = 9.57, *p* < 0.001, η^2^p = 0.27]. Similarly, amotivation exhibited a significant main effect of time [F(1, 52) = 10.26, *p* = 0.002, η^2^p = 0.16] and a significant group × time interaction [F(2, 52) = 13.01, *p* < 0.001, η^2^p = 0.33] (Table 2).

Post hoc analyses indicated that in the ADAPT + VID group, intrinsic motivation significantly increased from the first to the last session (*p* < 0.001), while amotivation significantly decreased (*p* < 0.001). In the ADAPT group, intrinsic motivation improved significantly over time (*p* = 0.01), but no significant changes in amotivation were observed. Identified regulation and external regulation did not show significant differences over time or between groups. At T1, the ADAPT + VID group reported significantly higher intrinsic motivation (*p* < 0.001) and lower amotivation (*p* = 0.002) than the control group.

## 4. Discussion

This study aimed to evaluate the effectiveness of an adapted basketball intervention program, enhanced using basketball skills demonstration videos, on the acquisition of basketball skills (i.e., passing, dribbling, and free throws), body mass index (BMI), physical fitness, and motivation in adolescents with obesity. The results of this multimodal approach were compared with those obtained with the same adapted basketball intervention, without the use of videos, and with those obtained after a conventional (without adapted) basketball program (CONT). The results showed that participants who used video support achieved significantly greater improvements in basketball technical skills, and motivation, compared to the other groups, and a modest but statistically significant improvement in BMI and physical fitness

The between-group analysis revealed that the ADAPT + VID group showed significantly superior technical performance in both passing and dribbling compared to the control group. This improvement highlights the effectiveness of video modeling in motor learning, in line with previous research showing that observing expert models facilitates the acquisition of motor patterns, especially among novices [30,42,43]. Several mechanisms may explain these technical advances.

Furthermore, observing a correct and competent model strengthens the mental representation of the movement, thereby facilitating its accurate motor reproduction [44,45,46]. Indeed, according to the socio-cognitive theory of observational learning [47], the observation of a stable model allows students to refine their execution.

Secondly, video viewing improves motor memory, improving the acquisition and retrieval of motor patterns [48]. Finally, the resulting reduction in cognitive effort allows students to devote more attention to postural adjustments and movement fluidity, thereby enhancing movement proficiency and self-esteem [49,50,51]. Furthermore, observing a competent model promotes a more confident attitude towards motor challenges [52].

Some studies have shown how observational learning improves perceived self-efficacy while reducing anxiety associated with the execution of complex motor tasks [53,54].

The increase in enthusiasm due to the reduction in performance-related stress determines a greater commitment to physical activity [55,56].

The increase in motivation is a very important result because, in general, overweight and adolescents with obesity showed higher scores on amotivation and regulation of physical activity, compared to their normal weight counterparts [57].

In contrast, adolescents with obesity and with high levels of internal motivation are more likely to adhere to a physical activity program to maintain weight loss, compared to their counterparts with low levels of intrinsic motivation [57,58,59] and internal motivation is essential for the long-term maintenance of benefits [60].

Finally, as suggested by Hills et al. [61], regular physical activity induces beneficial metabolic adaptations, such as increased basal metabolism and improved hormonal regulation, thus facilitating weight loss.

This motivational dynamic, related to regular participation in physical activity, has been shown to be effective in reducing body mass index (BMI). Although both experimental groups (ADAPT + VID and ADAPT) showed significant decreases in BMI, the decrease was significantly higher in the ADAPT + VID group. Weight management in adolescents with obesity represents an important public health problem. Our results are in line with previous studies that have shown that personalized and motivating physical activity programs allow for more effective weight regulation in adolescents with overweight or obesity [62,63,64,65].

Although weight loss is not necessary to achieve exercise-induced improvements in aerobic and anaerobic fitness [66], changes in body composition, particularly in obese individuals, may be linked to indices of aerobic fitness [67,68]. Our study also demonstrated that an 8-week adapted basketball program was effective in significantly increasing VO2max in students with obesity, in line with the available literature on adolescents with obesity, which has shown an increase in cardiorespiratory fitness following HIIT programs [69,70,71,72,73,74].

Concerning the program, since basketball is a high-intensity intermittent sport that resembles HIIT and incorporates some elements of strength training, our findings are consistent with previous studies on youth with obesity, which showed that combining training leads to significantly greater improvements in aerobic capacity [68,75].

These results, therefore, highlight the importance of interventions specifically designed for adolescents with obesity, who require approaches that simultaneously promote motor learning and psychological engagement.

Although this study provides valuable insights into the impact of video modeling within a physical education program adapted for adolescents with obesity, several limitations should be acknowledged. First, the relatively short duration of the 8-week intervention, while sufficient to produce significant improvements, does not allow for conclusions about the long-term sustainability of effects on BMI, motivation, and physical fitness. A longitudinal follow-up would be necessary to assess the persistence of these results. Second, although BMI is a widely used and practical indicator of weight status, it does not provide information on body composition (e.g., distribution of fat and lean mass). Future studies should incorporate more precise body composition assessments, such as bioelectrical impedance analysis, to allow a more detailed interpretation of the outcomes. Third, the sample size was relatively small, and recruitment was limited to a single school, which may affect the generalizability of findings to adolescents with obesity from different cultural or socio-economic backgrounds. Moreover, individual differences, such as baseline skill level, gender, previous sport experience, self-perception, or preferred learning modalities (e.g., visual, kinesthetic), may moderate the effects of video modeling and should be considered in future studies. Finally, no objective measures of motor learning or cognitive engagement (e.g., attention, concentration) were included, which could have strengthened the understanding of the mechanisms underlying the observed improvements.

Taken together, these limitations suggest that while the present findings are promising, further research with larger and more diverse samples, longer follow-up periods, and more comprehensive outcome measures is needed to confirm and extend these results.

## 5. Conclusions

This study demonstrates that integrating video modeling into adapted basketball training improves technical skills, motivation, and physical fitness among adolescents with obesity. These findings support the inclusion of video-based teaching strategies in physical education and sports programs to enhance skill acquisition and engagement. Future studies with larger and more diverse samples and longer follow-up periods are warranted to confirm the sustainability and generalizability of these benefits.

## Figures and Tables

**Table 1:** Structure of the basketball intervention program for students with obesity.

Week	Main Session Objective	CONT Group (Traditional Program)	ADAPT Group (Adapted Program)	ADAPT + VID Group (Adapted Program with Educational Videos)
**Week 1**	Discovery and familiarization	Introduction to basketball rules and basic techniques.	Same content, but alternating between low-intensity drills and fun, playful activities.	Same as ADAPT group, with prior viewing of a video summarizing rules and fundamental techniques.
**Weeks 2–3**	Development of individual skills	Standard 1-on-1 offensive and defensive drills.	Defender limited to one hand; may only engage after the attacker initiates movement.	Same as ADAPT group, preceded by an instructional video on passing, dribbling, and shooting.
**Weeks 4–5**	Reinforcement of team play	3-on-3 drills with standard offensive and defensive roles.	Drills modified to 3 attackers vs. 2 defenders (offensive advantage).	Same as ADAPT group, with a demonstrative video showing principles of attacking play in a 3v2 setup.
**Weeks 6–8**	Application in real game situations	5-on-5 matches with free substitutions based on students’ preferences.	5-on-5 matches with mandatory substitutions at each offensive transition.	Same as ADAPT group, with a summary video highlighting collective game principles in match situations.

**Table 2:** Changes in Situational Motivation valued before and after the intervention.

Motivational Dimensions	ADAPT + VID		ADAPT		CONTROL	
	T0	T1	T0	T1	T0	T1
**Intrinsic Motivation**	3.58 ± 1.43	5.26 ± 1.41 **^#^	3.56 ± 1.29	4.61 ± 1.33 *	3.44 ± 0.98	3.33 ± 1.46
**Identified Regulation**	3.84 ± 1.17	4.00 ± 1.45	3.50 ± 1.29	3.94 ± 1.16	3.78 ± 1	3.83 ± 1.20
**External Regulation**	3.79 ± 1.32	3.89 ± 1.20	3.83 ± 0.99	3.56 ± 1.15	3.67 ± 1.28	3.72 ± 1.07
**Amotivation**	4.11 ± 1.20	2.84 ± 1.07 **^#^	4.17 ± 0.99	3.56 ± 1.29	3.89 ± 1.02	4.39 ± 1.29

ADAPT + VID, adapted intervention group enriched with the prior delivery of video summaries of instructional content; ADAPT, adapted intervention group; *p* values adjusted for multiple testing by the Holm–Bonferroni method. Significant difference: * *p* < 0.05 vs. T0 within the same group; ** *p* < 0.01 vs. T0 within the same group; ^#^ *p* < 0.05 vs. CONTROL group at T1.

## Data Availability

The original contributions presented in the study are included in the article, further inquiries can be directed to the first author.
